# Mapping for maternal and newborn health: the distributions of women of childbearing age, pregnancies and births

**DOI:** 10.1186/1476-072X-13-2

**Published:** 2014-01-04

**Authors:** Andrew J Tatem, James Campbell, Maria Guerra-Arias, Luc de Bernis, Allisyn Moran, Zoë Matthews

**Affiliations:** 1 Department of Geography and Environment, University of Southampton, Highfield, Southampton, UK; 2 Fogarty International Center, National Institutes of Health, Bethesda, MD 20892, USA; 3 Instituto de Cooperación Social Integrare, Barcelona, Spain; 4 United Nations Population Fund, Geneva, Switzerland; 5 U.S. Agency for International Development, Washington DC, USA; 6 Department of Social Statistics and Demography, University of Southampton, Highfield, Southampton, UK

## Abstract

**Background:**

The health and survival of women and their new-born babies in low income countries has been a key priority in public health since the 1990s. However, basic planning data, such as numbers of pregnancies and births, remain difficult to obtain and information is also lacking on geographic access to key services, such as facilities with skilled health workers. For maternal and newborn health and survival, planning for safer births and healthier newborns could be improved by more accurate estimations of the distributions of women of childbearing age. Moreover, subnational estimates of projected future numbers of pregnancies are needed for more effective strategies on human resources and infrastructure, while there is a need to link information on pregnancies to better information on health facilities in districts and regions so that coverage of services can be assessed.

**Methods:**

This paper outlines demographic mapping methods based on freely available data for the production of high resolution datasets depicting estimates of numbers of people, women of childbearing age, live births and pregnancies, and distribution of comprehensive EmONC facilities in four large high burden countries: Afghanistan, Bangladesh, Ethiopia and Tanzania. Satellite derived maps of settlements and land cover were constructed and used to redistribute areal census counts to produce detailed maps of the distributions of women of childbearing age. Household survey data, UN statistics and other sources on growth rates, age specific fertility rates, live births, stillbirths and abortions were then integrated to convert the population distribution datasets to gridded estimates of births and pregnancies.

**Results and conclusions:**

These estimates, which can be produced for current, past or future years based on standard demographic projections, can provide the basis for strategic intelligence, planning services, and provide denominators for subnational indicators to track progress. The datasets produced are part of national midwifery workforce assessments conducted in collaboration with the respective Ministries of Health and the United Nations Population Fund (UNFPA) to identify disparities between population needs, health infrastructure and workforce supply. The datasets are available to the respective Ministries as part of the UNFPA programme to inform midwifery workforce planning and also publicly available through the WorldPop population mapping project.

## Background

Improved understanding of geographic variation and inequity in health status, wealth, and access to resources within countries is increasingly being recognized as central to meeting development goals. Approaches based on local epidemiological and coverage data have been identified as vital to reducing childhood mortality for Millennium Development Goal (MDG) 4 [1], while the sub-national heterogeneity in HIV [2,3] and malaria [4,5] prevalences mean that effective targeting of interventions remains vital in achieving MDG 6 [6]. Indicators assessed at national level can often conceal important inequities, with the rural poor often least well represented [7,8].

The fifth MDG, which targets maternal health, is widely recognised as being the most off-track of the goals endorsed by leaders in 2000 [9]. Moreover, with a little more than two years to go before the MDG deadline, maternal mortality is still the most dramatic indicator of global health inequity with 99% of these deaths occurring in developing countries, and large numbers likely going unrecorded. For every death, at least another 20 women suffer illness or injuries related to childbirth or pregnancy [10]. Mortality rates for newborn babies have also been slow to decline compared with death rates for older infants. Known solutions, such as the provision of a skilled health worker at births, are effective in tackling both maternal and newborn health problems, but scaling up services is still hampered by a lack of basic subnational data. The lack of spatial datasets to aid in identifying the magnitude of inequities for women and newborns both in outcomes and services are now a key constraint to progress [11,12]. Moreover, as international funding for health and development comes under pressure, the ability to target limited human resources and health services to underserved groups becomes crucial.

For maternal and newborn health (MNH) and survival there are three key sets of information that are currently lacking or poorly provided – that could be better estimated at small geographies to inform planning and policy and to pinpoint communities in need: (i) Planning for safer births and healthier newborns would be improved firstly by providing more accurate estimations of population distributions – targeting particularly women of childbearing age. (ii) Better projections of the approximate numbers of actual pregnancies that are likely to occur in the short and long-term are needed for more effective strategies on human resources and infrastructure. (iii) There is a need to link information on pregnancies to better information on health facilities in districts and regions so that distance to services and their coverage can be assessed. The latter is now becoming feasible given the move towards geo-referencing hospitals and health centres, and while there are many other factors that influence service utilisation, it should be a key indicator of progress given the importance of geographical access to services for the survival of women and newborns with complications [13,14]. The GIS techniques that can be applied to derive these three needed sets of estimations have been applied only sporadically in the area of maternal and newborn health [15]. Despite some progress in very recent years (e.g. [16]), it is recognised that there is insufficient global attention paid to disaggregating national data by geographical units [17] and the MNH community has yet to fully capitalize on the emerging capacity of GIS.

Here we present methods to estimate firstly women of childbearing age and secondly pregnancies and live births in relation to current health infrastructure in four countries (Afghanistan, Bangladesh, Ethiopia and Tanzania) with high maternal and neonatal mortality, and details on the MNH situation in each country are provided in the next section. The methods presented here are based on the integration of satellite, census, household and Emergency Obstetric and Newborn Care (EmONC) survey data for the construction of high resolution datasets depicting estimates and distribution of the number of people, women of childbearing age, pregnancies and live births per 100×100 m grid cell, plus locations of EmONC facilities. The application of these methods for constructing such datasets is presented, followed by a simple demonstration analysis in linking the likely distribution of need (pregnancies) compared with services to highlight geographic inequities in service provision.

### Study countries

#### Afghanistan

In Afghanistan, The Basic Package of Hospital Services (BPHS), revised in 2010 and Essential Package of Health Services (EPHS) created in 2005 have led to improvements in MNH indicators and services coverage. However, important gaps remain, as less than a third of deliveries take place in health facilities – just a fifth in the case of the most remote areas. Access to antenatal care (ANC) also exhibits wide geographical inequalities – ranging from 79% in the capital to 42% in the western region. Distance and lack of transport are perceived as important barriers to accessing these services. With the aim of expanding coverage, midwives have received significant policy support in the past decade, with the creation of a community midwife programme to rapidly train and deploy large numbers of these frontline health workers. Yet facility-based rather than population-based staffing guidelines mean that at the Basic Health Centre level there is only one skilled birth attendant (midwife) on staff, which presents a barrier to effective 24-hour coverage.

#### Ethiopia

In Ethiopia, there are huge inequities in skilled birth attendance, with rates ranging from 51% in urban areas to 4% in rural areas. In certain regions, over half of all births are attended by traditional birth attendants. There are also huge wealth-based disparities, with the poorest women far less likely to access skilled birth attendants (SBA) and ANC, or to use contraception. However, despite, the regional disparities, midwives and health workers are quite equitably distributed within rural areas. The workload per health worker is very high – the average ratio of midwives to pregnancies is 1:1,159. Government policy aims to increase skilled birth attendance and scale up the provision of Basic and Comprehensive Emergency Obstetric and Neonatal Care (BEmONC and CEmONC), with health worker targets set by the government based on staffing standards per health facility. Despite scaling up facilities and workforce numbers, inadequate transportation networks and cultural perceptions still result in low numbers of pregnant women accessing MNH care.

#### Bangladesh

The level of skilled birth attendance in Bangladesh is low (32%), with great disparities between coverage in urban areas (54%) and rural areas (25%) – despite the fact that 78% of pregnant women lived in rural areas in 2010. A huge challenge is inequitable distribution of skilled health professionals: up to half of the country’s nurse-midwives and doctors are located in the district of Dhaka, which has just 8% of the nation’s pregnancies. National policies have focused on promoting institutional deliveries and skilled attendance at birth, and increasing the number and quality of EmONC facilities. However, progress towards these goals has been slow. Retaining health workers in undesirable rural posts remains an issue, and levels of health worker absenteeism are very high.

#### Tanzania

In Tanzania, there is a strong policy focus on reducing maternal and newborn mortality rates, yet these are declining slowly and off-track to meet the MDGs. Regional inequalities are extremely large: facility-based deliveries rates vary from 30% to 90% in the highest and lowest scoring districts. In total 50% of the births in Tanzania take place outside of facilities, with a strong preference for relying on traditional health practitioners. There is no one specific health worker cadre competent in the full set of midwifery competencies and dedicated to frontline maternal and newborn care. Instead, different health cadres such as enrolled nurses, medical officers, clinical officers, community health workers and others carry out different essential interventions along the MNH continuum of care. A key strategy of the Ministry of Health and Social Welfare is the strengthening of recruitment, retention and deployment of health workers. In recent years, there have been efforts to double annual enrolment in training institutions, particularly for enrolled nurses, clinical officers and assistant clinical officers. Another measure is the provision of incentives for postings in rural areas.

## Methods

The methods and calculations originate from health systems and policy research in the four countries, undertaken as part of the High Burden Countries Initiative (HBCI) supported by UNFPA and its UN partners in the H4+ [18]. As part of their broader responses to the UN Secretary-General’s Global Strategy for Women’s and Children’s Health, the UN health agencies - “H4+” (UNAIDS, UNFPA, UNICEF, World Bank, WHO), alongside the government and development partners, have initiated national assessments of the midwifery workforce in 8 countries (Afghanistan, Bangladesh, Democratic Republic of Congo, Ethiopia, India, Mozambique, Nigeria and the United Republic of Tanzania), representing nearly 60% of the global maternal and newborn deaths. All four countries are listed amongst the 10 countries with the highest burden of maternal and neonatal deaths per annum [19-21]. The work here describes the development of the methods needed in preparation for a larger midwifery workforce assessment that seeks to generate new evidence on ‘w*hat is the appropriate midwifery workforce, and how is it best deployed, to equitably deliver essential MNH interventions at scale and quality, and what (including costs) needs to be put into place to achieve universal access?’* Each of the country assessments follows an Operational Guidance and Assessment Framework [19] and includes a module on the ‘Geography of MNH’ to estimate and map where women of child-bearing age are living and how many pregnancies are likely to be occurring. This provides a proxy for population need for MNH services that can ultimately be linked with georeferenced facility data to compare the current supply-side capacity of health facilities and workforce deployment to identify inequities in access to midwifery care and services.

In order to complete the Geography of MNH module we developed new methods using a geographical information system (GIS) based approach, enabling the construction and combination of spatial data layers. Firstly we construct population distributions at subnational level and provide estimates of women of reproductive age per grid cell. We then estimate numbers of pregnancies and live births for each grid cell using national household survey data on age specific fertility rates, published estimates of live births from the UN Population Division [22] and new estimates of stillbirths, miscarriages and abortions from the Guttmacher Institute [23]. A third layer of the geographical location of health facilities providing basic and comprehensive emergency obstetric and newborn care is then added. Finally we demonstrate the utility of these layered datasets in estimating proximities from pregnancies to health facilities – linking estimates of population need with the locations of facilities designed to meet this need.

### Constructing detailed and contemporary population distribution datasets

The WorldPop project (http://www.worldpop.org.uk), has recently completed construction of 2010 estimates of population distribution for continental Africa plus Madagascar [24] and Southeast and Central Asia [25] at approximately 100 m spatial resolution. Full details are provided on the project websites, along with links to papers describing the methods in detail [24-30]. Briefly, a GIS-linked database of census and official population estimate data was constructed, targeting the most recent and spatially detailed datasets available, given their importance in producing accurate mapping. Detailed 30 metre spatial resolution maps of settlement extents were derived from Landsat satellite imagery through either semi-automated classification approaches [28-30] or expert opinion-based analyses. These settlement maps were then used to refine land cover data, incorporating the improved settlement outlines into the data. Meanwhile, local census data mapped at enumeration area level from sample countries across Africa and Asia were exploited to identify typical regional per-land cover class population densities. These densities were then used to redistribute census counts, stratified by regional ecozones, to map human population distributions at approximately 100 m spatial resolution continent-wide. Where available, additional country-specific datasets providing valuable data on population distributions not captured by censuses, such as internally displaced people or detailed national surveys, were incorporated into the mapping process.

UN estimates of urban and rural-specific growth rates by 5-year periods under low, medium and high scenarios [31] were compiled for all African and Asian countries. These rates were applied to the 2010 datasets described above, with the urban rates applied to those mapped urban areas that fell within the extents of Columbia University’s Global Rural Urban Mapping Project (GRUMP) urban extent map [32]. The rural rates were applied elsewhere. This approach was used to construct 2012 population distribution datasets, which were adjusted to ensure that national population totals matched those estimated by the UN under their low, medium and high scenarios. For the remainder of this paper, we focus on outputs using the medium scenarios.

Tatem et al. [33] describes how data on sub-national population compositions were obtained from a variety of sources for all African countries, principally from contemporary census-based counts broken down at a fine resolution administrative unit level. These were matched to corresponding GIS datasets showing the boundaries of each unit, and used to adjust the existing WorldPop spatial population datasets described above to produce estimates of the distributions of populations by sex and five-year age group. The datasets were then adjusted to ensure that national population totals by age group, specific city totals and urban/rural totals matched those reported by the UN [22]. Here, this process was also undertaken for Bangladesh and Afghanistan, using 2011 Upazila level census data for Bangladesh and 2010 Demographic and Health Survey (DHS) program data for Afghanistan to produce estimates of the distributions of populations by sex and five-year age group. For each of the four study countries, summation of the datasets representing females in the 15–49 year age groups was undertaken to produce women of childbearing age datasets.

### Mapping pregnancies and live births

For the four study countries, age-specific fertility rates (ASFRs) by 5-year age groupings disaggregated by subnational regions and urban versus rural were derived from the most recent national household surveys conducted as part of the DHS programme (http://www.measuredhs.com). This involved 8 subregions for Afghanistan from the 2010 survey, 7 subregions for Bangladesh from the 2011 survey, 11 subregions for Ethiopia from the 2011 survey and 26 subregions for Tanzania from the 2010 survey. Each set of subregions were broken down further by urban and rural areas. The subnational ASFRs were estimated using a Stata program developed by Pullum [34]. ASFRs were calculated by dividing the number of births to women in a specified age group during a specified time period by the number of woman-years of exposure during the same period. The ASFRs correspond to women for the seven five-year age groups from 15–19 to 45–49. For current fertility rates, the Demographic and Health Surveys (DHS) use the period 1–36 months (three years) before the survey, and the sample sizes in Afghanistan, Tanzania and Ethiopia included all women, while for Bangladesh it was ever-married women. GIS datasets representing the boundaries of the subregions (http://www.measuredhs.com/What-We-Do/GIS.cfm) and the urban extents [32] within them were assembled, and the ASFRs matched to them. These rates were then used to adjust each 5-year age grouped female population distribution dataset described above to produce gridded estimates of the distributions of live births across each study country. The national totals were then adjusted to match those live birth totals estimated by the Guttmacher Institute for 2012 [23]. Finally, to convert these gridded datasets of numbers of live births to numbers of pregnancies for each of the four study countries, the national totals were adjusted to match national estimates of numbers of pregnancies [23].

### Quantifying the proximity of pregnancies to EmONC facilities

Where comprehensive data exist on health facility locations and their functions and features (such as signal functions, staffing levels, infrastructure, case fatality rates, intrapartum stillbirth rates and rates of interventions), e.g. through an EmONC survey, these can be used in combination with the datasets on mapped pregnancies described above to estimate numbers of pregnancies within user-defined distances or travel times of a CEmONC facility, or user-defined definitions of facilities. Many previous studies have measured, mapped and modelled travel times to health facilities using a range of different approaches, levels of complexity and assumptions (e.g. [16,35-37]). Here we undertook a simple illustrative analysis for Ethiopia, as the additional dataset assembly, empirical travel time weighting derivations and model construction were beyond the scope of this study. Data on CEmONC facilities with GPS locations were extracted from the national EmONC survey and first converted to GIS shapefile format, then reprojected to Mollweide projection (an equal area map projection, appropriate for calculating distances). Following this, buffers of 50 km radii around each facility were mapped (50 km being a rough proxy for 2 hours travel time by motorized transport). These buffer zones were then overlaid on the Ethiopia pregnancies dataset described above, and numbers within the 50 km buffers calculated. Similarly, numbers residing outside of these buffers were also calculated, and for each administrative level 3 unit (woreda), the percentage of pregnancies that fell within 50 km of a CEmONC facility were calculated and mapped.

## Results

### Constructing detailed and contemporary population distribution datasets

**Figure 1:**
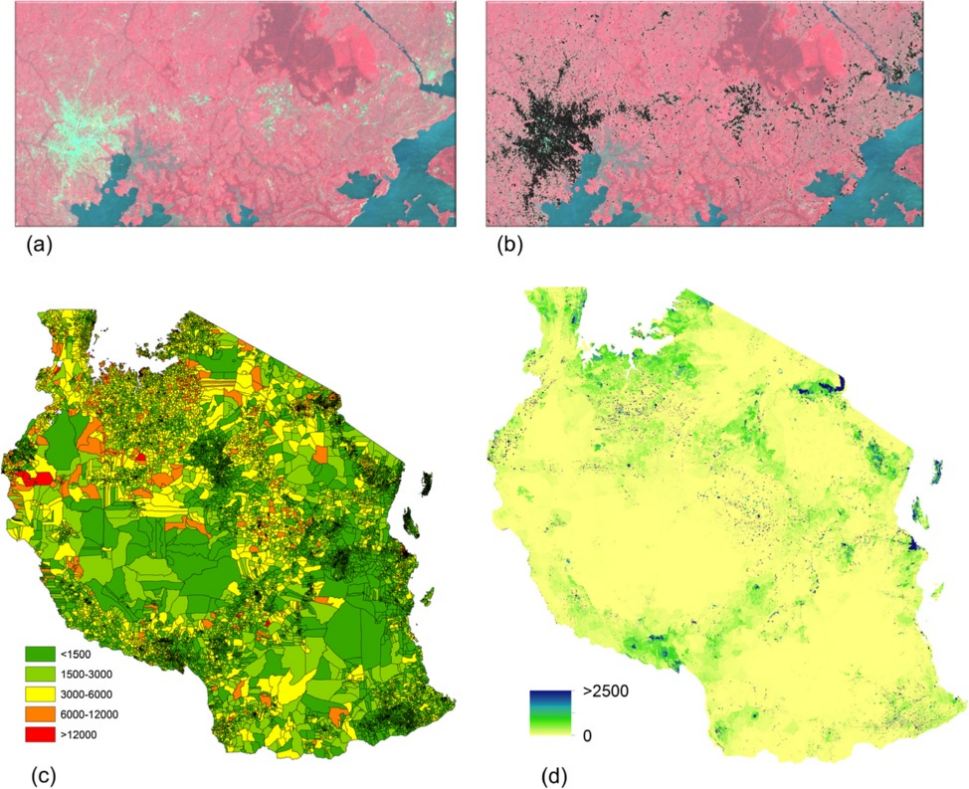
**Mapping settlements and population distribution. (a)** Landsat Enhanced Thematic Mapper (ETM) image showing a city and surrounding smaller settlements; **(b)** Settlements extracted using automated mapping approach; **(c)** Census data for Tanzania in 2002 at village level showing total number of people per unit; **(d)** Population distribution map for 2012 produced through using the satellite derived settlement and land cover information to reallocate census unit populations to a 100×100 m resolution grid, followed by application of UN urban/rural growth rates.

**Figure 2:**
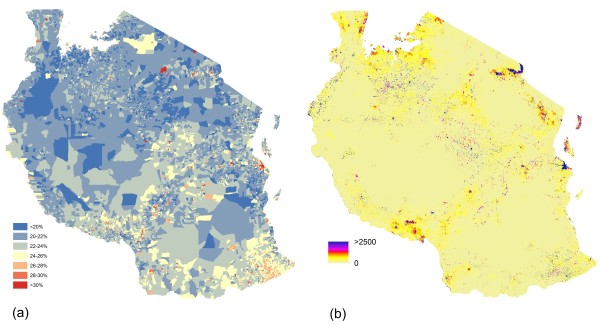
**Mapping women of childbearing age. (a)** Proportion of the population that are women of childbearing age (15-49 yrs) from village-level census data; **(b)** Population distribution map showing estimated number of women of childbearing age per 100×100 m grid cell.

Population distribution datasets were constructed for Afghanistan, Bangladesh, Ethiopia and Tanzania, and Figure 1 presents examples of the datasets and outputs for Tanzania. Figure 1a shows the original Landsat imagery, and Figure 1b shows the extracted areas of human settlement. These settlement data were then combined with the detailed administrative boundary linked census count data (shows in Figure 1c) and land cover data to produce the gridded datasets of estimated numbers of people per 100×100 m grid cell (Figure 1d). Finally, the administrative boundary linked census data on proportions of people in each 5-year age grouping (0–4, 5–9, 10–14 etc.) by sex were used to adjust the gridded population datasets to obtain age-structured population datasets, and Figure 2 shows the census counts (Figure 2a) and gridded dataset (Figure 2b) for women of child bearing age in Tanzania.

### Mapping pregnancies and live births

**Figure 3:**
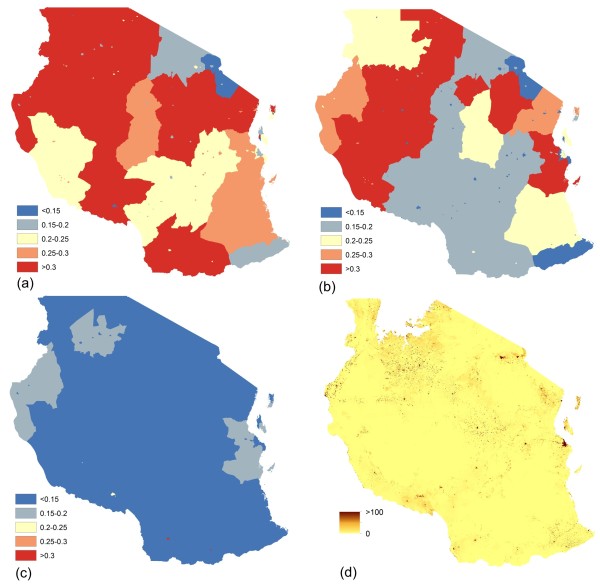
**Age-specific fertility rates and mapped live births. (a)** Fertility rates for women aged 20-24 mapped in five categories and constructed from the 2010 Tanzania Demographic and Health Survey; **(b)** Fertility rates for women aged 30-34 mapped in five categories and constructed from the 2010 Tanzania Demographic and Health Survey; **(c)** Fertility rates for women aged 40-44 mapped in five categories and constructed from the 2010 Tanzania Demographic and Health Survey; **(d)** Map showing estimated total number of live births per 100×100 m grid cell in 2012.

**Figure 4:**
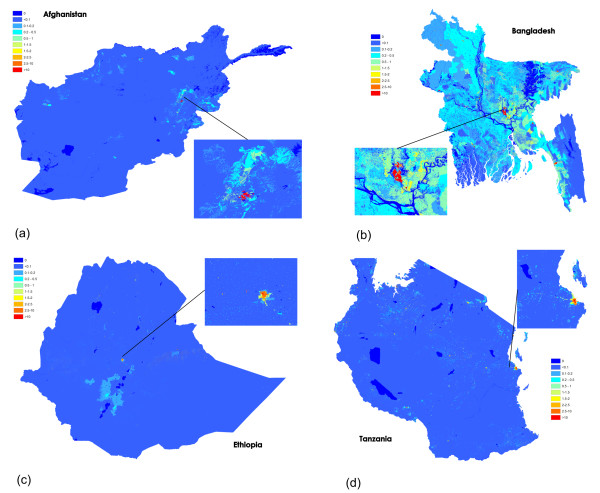
**Gridded datasets of estimated numbers of pregnancies per 100×100 m grid cell for 2012** for **(a)** Afghanistan, with close-up of Kabul area shown; **(b)** Bangladesh, with close-up of Dhaka area shown; **(c)** Tanzania, with close-up of Dar Es Salaam and Zanzibar area shown; **(d)** Ethiopia, with close-up of Addis Ababa area shown.

Following construction of the age-structured population datasets, the DHS-derived subnational urban/rural age-specific fertility rates (ASFRs) were applied to convert these to estimated live births datasets. Figures 3a-c highlight the substantial differences that exist between age groups and across Tanzania in terms of ASFRs, and the need therefore to account for such differences in defining subnational estimates of numbers of live births. The application of these ASFRs to the age and sex structured gridded population datasets resulted in estimates of numbers of live births in total per 100×100 m grid cell (Figure 3d). Finally, the birth datasets were adjusted to produce per grid cell estimates of numbers of pregnancies in 2012. These are presented for the four study countries in Figure 4.

### Quantifying the proximity of pregnancies to CEmONC facilities

**Figure 5:**
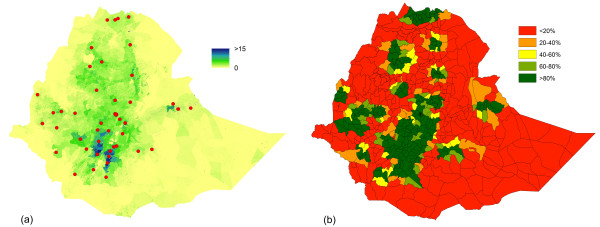
**Quantifying the proximity of pregnancies to CEmONC facilities. (a)** Dataset illustrating the estimated number of pregnancies per 100×100 m grid cell on a standard deviation colour scale in 2012 for Ethiopia, with the location of comprehensive EmONC facilities overlaid; **(b)** Estimated percentage of pregnancies within 50km of a comprehensive EmONC facility in Ethiopia in 2012 by woreda administrative unit.

Figure 5 shows the results of a simple distance-based analysis of facility provision in Ethiopia. Figure 5a shows the estimated number of pregnancies per grid cell in Ethiopia in 2010 with the location of CEmONC facilities overlaid. By applying 50 km buffers to these facility locations and summing the estimated number of pregnancies within them, a simple analysis on the proximity of pregnancies to facilities was undertaken, with the percentage of pregnancies within each woreda that were within 50 km of a CEmONC facility shown in Figure 5b, highlighting geographical differences in coverage. This highlights the potential risk to women and babies who are more than two-hours travel (by motorised transport) from access to life-saving interventions.

## Discussion

Mapping and the application of GIS in many health related fields is becoming sophisticated and demonstrated as a valuable tool for providing evidence to guide strategies, but its application in MNH is lagging behind. The midwifery workforce assessments sought to integrate the capacity and added value of GIS technology and the approaches outlined here for population, age group, pregnancy, birth and facility mapping to produce gridded surfaces, with each 100 m by 100 m grid cell providing an estimate of the number of people, live births, and pregnancies within it. Such datasets provide exceptional flexibility in terms of enabling summarization to any level required. This may include, for example: (i) summing all grid square estimates within district boundaries to provide district-level estimates of number of pregnancies, (ii) usage of a map of urban areas overlaid onto the gridded data to provide per-city estimates of number of pregnancies, or urban rural proportions, and (iii) integration of the gridded datasets with health facility locations and road network data within travel models to quantify numbers of pregnant women residing at more than two hours from a comprehensive EmONC facility. In each of these example cases, simple national averages that do not account for subnational geography cannot reveal the spatial heterogeneities that exist in this way.

While it has been shown that accounting for sub-national heterogeneity in population attributes likely results in significant improvements in the accuracy of health metrics [33], it is clear that many sources of uncertainty and error remain. Primarily, it is clear that uncertainties in the output maps increase and accumulate at each stage of the process. While the location and number of total people and women of childbearing age subgroup are known with a relatively good degree of precision, the subnational variations in birth rates are less precisely known, while subnational information on pregnancies, abortions and stillbirths do not currently exist. This means for pregnancies, a reliance on the assumption of no spatial variation within a country on rates of stillbirths and abortions through the use of national-level estimates [23]. Further, all of the census and survey-based data used here are subject to various sources of error and bias, many of which have been well-documented [33,38,39], while the underlying WorldPop population datasets also contain uncertainties [24]. It is also clear that the sampling frames used in undertaking the DHS surveys mean that uncertainties exist in defining sub-national urban/rural ASFRs, with sample sizes becoming relatively small when summarizing at such levels. Nevertheless, the resultant ASFRs do still match closely with what is known about regional and urban–rural differences in each of the countries, with lower rates in urban areas and the highest rates in the most isolated and deprived rural areas of each country (Figure 3). Like most other population parameters reported for administrative polygons, the population, age, sex and fertility rate data used here are also subject to the modifiable areal unit problem, i.e. that summary measures are influenced by the administrative boundaries that they are reported at [40]. Finally, as highlighted in the methods section, assessments of geographical access to care could be improved through the modelling of travel times, rather than simple straight-line distances.

There is clearly a need to more rigorously quantify the uncertainties inherent in spatial demographic datasets [39], such as those presented here to better communicate the spatial variations in reliability of input datasets and guide prioritization of additional data collection, and future work will aim to tackle this. The advancement of theory, increasing availability of computation, and growing recognition of the importance of robust handling of uncertainty have all contributed to the emergence in recent years of new, sophisticated approaches to the large-scale modeling and mapping of disease based on geostatistics (e.g. [4]) and small area estimation [41], but such methods have yet to cross over to the spatial demographic databases with which such maps are used. The regular availability of new national household surveys means that more contemporary data is continually becoming available to aid in updating and improving the accuracy of the datasets presented here, and future steps will involve the development of semi-automated systems that can rapidly adapt to new incoming data and integrate them into the output spatial datasets, alongside robust methods to account for temporal differences [42]. Further, the linkage of these pregnancy datasets with the location of health facilities and spatial models of travel time will enable improved estimates of the spatial coverage of healthcare to be made.

Despite the limitations and caveats above the results in all four countries provide new intelligence on disaggregated population needs for MNH services and are informing the policy discourses on the distribution of health facilities and health personnel who provide MNH/midwifery services. However, not all countries maintain an accurate, current list of EmONC facilities with georeferenced codes and even fewer maintain a live database on the number, type, competency and skill levels of health personnel per facility. The absence of this basic information therefore diminishes the ability to conduct detailed analysis of supply-side constraints to respond to population need. Improvements in human resource information systems are critically needed and if linked to facility GIS codes would lead to new insights on accessibility to a skilled and competent health worker. Additionally we recognise that comparing need to supply means that we are missing an important step: women’s demand for and utilisation of MNH/midwifery services. In all four countries, coverage of antenatal care, skilled attendance at birth and postnatal care is variable, with significant difference between urban/rural areas and socio-economic quintiles. Barriers to access care are therefore beyond the geographical location of services and require triangulation with other sources of data to appreciate the realities that women experience in seeking, accessing and affording quality care during pregnancy, birth and the post-natal periods.

## Conclusions

Thus far, the measurement of health outcomes and service needs has focussed on indicators at country level for member states that signed up to the original MDG goals. But increasingly there are concerns that even where progress has been made, this is subject to very wide inequalities. This means that countries that have progressed well include subgroups in their populations where survival rates and access to services have not changed – or have even worsened. Investigation of subnational situations is therefore needed and a geographical analysis is now increasingly required. An improved understanding of geographic variation and inequity in health status, wealth, and access to resources within countries is increasingly being recognized as central to meeting development goals.

Substantial demographic variations exist across countries and between urban and rural areas [39]. With MDG health indicators tied to specific vulnerable groups, there is a need to know who and where these vulnerable groups are and the numbers of individuals at risk that exist in order to accurately characterize denominators. High fertility is still a feature of many high burden countries and in rural outlying areas where resources are stretched, stalled fertility declines and sheer population momentum mean that an increasing number of births need to be attended. Though facility workloads are affected by many factors, clearly there are localities where increasing numbers of pregnancies and births have not been matched by commensurate increases in the availability of the appropriate MNH workforce, leading to a deterioration of the MNH situation even while the national picture may have been improving. In recent years tracking results and resources in sub national areas are not only more possible but also more necessary.

The methods presented in this paper, notwithstanding their uncertainties, represent a step forward in terms of estimation that can help to quantify progress and problems at very small geographies. The initial work in support of the national midwifery workforce assessments in Afghanistan, Bangladesh, Ethiopia and Tanzania has established the potential value to inform policy-dialogue on geographical gaps in service provision in response to population needs [43]. We envisage that such methods will increasingly inform international and national level evidence-based decision-making where denominators are uncertain, i.e. where civil registration and vital statistics of births and deaths are poor or patchy, and GIS-based techniques such as these are an important part of the data revolution to assist policymakers to improve services and survival. The live birth and pregnancy datasets outlined in this paper are freely available through the WorldPop project website (http://www.worldpop.org.uk).

## Competing interests

The authors declare that they have no competing interests.

## Authors’ contributions

AJT conceived the analyses, undertook the data assembly, mapping and processing and contributed to writing the manuscript. JC provided guidance in designing analyses and contributed to writing the manuscript. MG-A provided input datasets and contributed to writing the manuscript. LB provided guidance in designing analyses and contributed to writing the manuscript. AM contributed to writing the manuscript. ZM provided guidance in designing analyses, contributed input data and contributed to writing the manuscript. All authors read and approved the final manuscript.

## References

[B1] Kinney MV Kerber KJ Black RE Cohen B Nkrumah F Coovadia H Nampala PM Lawn JE Axelson H Bergh AM Sub-Saharan Africa's mothers, newborns, and children: where and why do they die? PLoS Med 2010 7 e1000294 10.1371/journal.pmed.1000294 20574524 PMC2888581

[B2] Kleinschmidt I Pettifor A Morris N MacPhail C Rees H Geographic distribution of human immunodeficiency virus in South Africa Am J Trop Med Hyg 2007 77 1163 1169 18165541 PMC3836237

[B3] UNAIDS Global Report: UNAIDS report on the global AIDS epidemic 2010 Geneva, Switzerland: UNAIDS

[B4] Gething PW Patil AP Smith DL Guerra CA Elyazar IR Johnston GL Tatem AJ Hay SI A new world malaria map: Plasmodium falciparum endemicity in 2010 Malar J 2011 10 378 10.1186/1475-2875-10-378 22185615 PMC3274487

[B5] Snow RW Amratia P Kabaria CW Noor AM Marsh K The changing limits and incidence of malaria in Africa: 1939–2009 Adv Parasitol 2012 78 169 262 22520443 10.1016/B978-0-12-394303-3.00010-4 PMC3521063

[B6] United Nations Millenium Declaration http://www.un.org/millennium/declaration/ares552e.pdf

[B7] Gwatkin DR How much would poor people gain from faster progress towards the Millennium Development Goals for health? Lancet 2005 365 813 817 15733726 10.1016/S0140-6736(05)17992-6

[B8] Reidpath DD Morel CM Mecaskey JW Allotey P The Millennium Development Goals fail poor children: the case for equity-adjusted measures PLoS Med 2009 6 e1000062 10.1371/journal.pmed.1000062 19399155 PMC2667271

[B9] International Planned Parenthood Foundation and Action for Global Health MDG5 Improve Maternal Health 2010 Tenerife, Spain: Parliamentary Assembly, IPPF

[B10] UNICEF The state of the world's children 2009: maternal and newborn health 2008 New York: UNICEF

[B11] Neal SE Matthews Z Investigating the role of health care at birth on inequalities in neonatal survival: evidence from Bangladesh Int J Equity Health 2013 12 17 10.1186/1475-9276-12-17 23496964 PMC3606405

[B12] Bell GW Fitzmaurice J Neal SE Qomariyah SN Matthews Z Kehoe S, Neilson J, Norman J The geography of maternal death Maternal and infant deaths: Chasing Millennium Development Goals 4 and 5 2010 London: Royal College of Obstetricians and Gynaecologists 3 29

[B13] Gabrysch S Campbell OM Still too far to walk: literature review of the determinants of delivery service use BMC Pregnancy Childbirth 2009 9 34 10.1186/1471-2393-9-34 19671156 PMC2744662

[B14] Murray SF Pearson SC Maternity referral systems in developing countries: current knowledge and future research needs Soc Sci Med 2006 62 2205 2215 10.1016/j.socscimed.2005.10.025 16330139

[B15] Ebener S Guerra-Arias M Campbell J Tatem AJ Moran A Amoako Johnson F Fogstad H Stenberg K Neal SE Bailey P The geography of maternal and newborn health: the state of the art 2013 Bangkok, Thailand: Health GIS 10.1186/s12942-015-0012-x PMC4453214 26014352

[B16] Gething PW Johnson FA Frempong-Ainguah F Nyarko P Baschieri A Aboagye P Falkingham J Matthews Z Atkinson PM Geographical access to care at birth in Ghana: a barrier to safe motherhood BMC Public Health 2012 12 991 10.1186/1471-2458-12-991 23158554 PMC3533981

[B17] Bhutta ZA Global child survival: beyond numbers Lancet 2012 379 2126 2128 10.1016/S0140-6736(12)60686-2 22579124

[B18] World Health Organization H4+ High Burden Country Initiative (HBCI) 2013 Geneva: WHO

[B19] HBCI Secretariat and Technical Working Group H4+ High Burden Countries Initiative. National assessments - midwifery workforce: operational guidance and assessment framework 2012 Geneva: WHO

[B20] World Health Organization Trends in maternal mortality: 1990 to 2010 2012 Geneva: WHO

[B21] United Nations Inter-agency group for child mortality estimation Levels and trends in child mortality: Report 2012 2012 New York: UNICEF 10.1371/journal.pone.0101112 PMC4094389 25013954

[B22] United Nations Population Division World population prospects, 2012 revision 2012 New York: United Nations

[B23] Singh S Darroch JE Ashford LS Adding it up: the need for and cost of maternal and newborn care - estimates for 2012 2013 New York, USA: Guttmacher Institute

[B24] Linard C Gilbert M Snow RW Noor AM Tatem AJ Population distribution, settlement patterns and accessibility across Africa in 2010 PLoS One 2012 7 e31743 10.1371/journal.pone.0031743 22363717 PMC3283664

[B25] Gaughan AE Stevens FR Linard C Jia P Tatem AJ High resolution population distribution maps for Southeast Asia in 2010 and 2015 PLoS One 2013 8 e55882 10.1371/journal.pone.0055882 23418469 PMC3572178

[B26] Linard C Alegana VA Noor AM Snow RW Tatem AJ A high resolution spatial population database of Somalia for disease risk mapping Int J Health Geogr 2010 9 45 10.1186/1476-072X-9-45 20840751 PMC2949749

[B27] Linard C Gilbert M Tatem AJ Assessing the use of global land cover data for guiding large area population distribution modelling Geo J 2010 doi:10.1007/s10708-010-9364-8 10.1007/s10708-010-9364-8 PMC3617592 23576839

[B28] Tatem AJ Noor AM Hay SI Defining approaches to settlement mapping for public health management in Kenya using medium spatial resolution satellite imagery Rem Sens Env 2004 93 42 52 10.1016/j.rse.2004.06.014 PMC3350067 22581984

[B29] Tatem AJ Noor AM Hay SI Assessing the accuracy of satellite derived global and national urban maps in Kenya Remote Sensing Environ 2005 96 87 97 10.1016/j.rse.2005.02.001 PMC3350068 22581985

[B30] Tatem AJ Noor AM von Hagen C di Gregorio A Hay SI High resolution population maps for low income nations: combining land cover and census in East Africa PLoS One 2007 2 e1298 10.1371/journal.pone.0001298 18074022 PMC2110897

[B31] United Nations Population Division World urbanization prospects, 2011 revision 2011 New York: United Nations

[B32] Balk DL Deichmann U Yetman G Pozzi F Hay SI Nelson A Determining global population distribution: methods, applications and data Adv Parasitol 2006 62 119 156 16647969 10.1016/S0065-308X(05)62004-0 PMC3154651

[B33] Tatem AJ Garcia AJ Snow RW Noor AM Gaughan AE Gilbert M Linard C Millennium development health metrics: where do Africa’s children and women of child bearing age live? Popul Health Metrics 2013 11 11 10.1186/1478-7954-11-11 PMC3724578 23875684

[B34] Pullum T Program to produce ASFRs, TFR, GFR for specific windows of time, with covariates Pers Commun 2012

[B35] Blanford JI Kumar S Luo W Maceachren AM It's a long, long walk: accessibility to hospitals, maternity and integrated health centers in Niger Int J Health Geogr 2012 11 24 10.1186/1476-072X-11-24 22737990 PMC3515413

[B36] Huerta Munoz U Kallestal C Geographical accessibility and spatial coverage modeling of the primary health care network in the Western Province of Rwanda Int J Health Geogr 2012 11 40 10.1186/1476-072X-11-40 22984920 PMC3517388

[B37] Alegana VA Wright JA Pentrina U Noor AM Snow RW Atkinson PM Spatial modelling of healthcare utilisation for treatment of fever in Namibia Int J Health Geogr 2012 11 6 10.1186/1476-072X-11-6 22336441 PMC3292929

[B38] Hobcraft J McDonald J Rutstein SO Socioeconomic factors in infant and child mortality: a cross-national perspective Pop Studies 1984 38 193 223 11621399

[B39] Tatem AJ Adamo S Bharti N Burgert CR Castro M Dorelien A Fink G Linard C Mendelsohn J Montana L Mapping populations at risk: Improving spatial demographic data for infectious disease modeling and metric derivation Pop Health Metrics 2012 in press 10.1186/1478-7954-10-8 PMC3487779 22591595

[B40] Openshaw S The Modifiable Areal Unit Problem 1984 Norwich: Geo Books

[B41] Lawson AB Bayesian Disease Mapping: Hierarchical modeling in spatial epidemiology 2009 Boca Raton, FL, USA: CRC Press

[B42] Raftery AE Li N Sevcikova H Gerland P Heilig GK Bayesian probabilistic population projections for all countries Proc Natl Acad Sci U S A 2012 109 13915 13921 10.1073/pnas.1211452109 22908249 PMC3435191

[B43] Instituto de Coperacion Social Integrare H4+ High Burden Countries Initiative: Country Updates 2013 Barcelona: Integrare Available at: http://integrare.es/h4-high-burden-countries-initiative-the-reports/

